# Asiaticoside Attenuates Cell Growth Inhibition and Apoptosis Induced by Aβ_1-42_ via Inhibiting the TLR4/NF-κB Signaling Pathway in Human Brain Microvascular Endothelial Cells

**DOI:** 10.3389/fphar.2018.00028

**Published:** 2018-01-30

**Authors:** Daqiang Song, Xian Jiang, Yiliu Liu, Yuhong Sun, Shousong Cao, Zhuo Zhang

**Affiliations:** ^1^Department of Pharmacology, Southwest Medical University, Luzhou, China; ^2^Department of Anesthesiology, The Affiliated Hospital of Southwest Medical University, Luzhou, China

**Keywords:** Alzheimer’s disease, asiaticoside, human brain microvascular endothelial cells (hBMECs), Aβ_1-42_, TLR4, NF-κB

## Abstract

Alzheimer’s disease (AD) is a very common progressive neurodegenerative disorder with the highest incidence in the world. Dysfunction of the blood–brain barrier (BBB) may be responsible for the pathogenesis and pathology of AD for abnormally transporting amyloid-β (Aβ, the main component of the senile plaques) from the sera into the central nervous system. Aβ peptides induce apoptosis in human brain microvascular endothelial cells (hBMECs), the main component of BBB. Apoptosis in neuronal cells plays a critical role in the pathogenesis of AD. Asiaticoside, a natural glycoside extracted from Centella asiatica (L.) Urban, has an anti-apoptotic effect on hBMECs but the molecule mechanism remains unclear. Therefore, we investigate the protective effect of asiaticoside on Aβ_1-42_-induced cytotoxicity and apoptosis as well as associated mechanism in hBMECs with commonly used *in vitro* methods for clinical development of asiaticoside as a novel anti-AD agent. In the present study, we investigated the effects of asiaticoside on cytotoxicity by Cell Counting Kit-8 assay, mitochondrial membrane potential by JC-1 fluorescence analysis, anti-apoptosis by Hoechst 33258 staining and Annexin V-FITC (fluorescein isothiocyanate) and propidium iodide (PI) analyses, the expressions of TNF-α and IL-6 by enzyme-linked immunosorbent assay (ELISA) and TLR4, MyD88, TRAF6, p-NF-κB p65, and total NF-κB p65 by Western blotting, and nuclear translocation of NF-κB p65 by immunofluorescence analysis in hBMECs. The results showed that pretreatment of asiaticoside (25, 50, and 100 μM) for 12 h significantly attenuated cell growth inhibition and apoptosis, and restored declined mitochondrial membrane potential induced by Aβ_1-42_ (50 μM) in hBMECs. Asiaticoside also significantly downregulated the elevated expressions of TNF-α, IL-6, TLR4, MyD88, TRAF6, and p-NF-κB p65, as well as inhibited NF-κB p65 translocation from cytoplasm to nucleus induced by Aβ_1-42_ in hBMECs in a concentration-dependent manner. The possible underlying molecular mechanism of asiaticoside may be through inhibiting the TLR4/NF-κB signaling pathway. Therefore, asiaticoside may be developed as a novel agent for the prevention and/or treatment of AD clinically.

## Introduction

Alzheimer’s disease (AD) is a common neurodegenerative disorder characterized by selective and progressive loss of specific neuronal populations in the neocortex and hippocampus (Pacheco-Quinto et al., 2016). Currently, accumulated evidence has shown that the major pathological causes of AD are the abnormal extracellular deposition of senile plaques (SPs) mainly containing amyloid-β (Aβ) protein and intracellular accumulation of neurofibrillary tangles (NFTs) primarily formed by hyperphosphorylated tau in the brain (Metcalfe and Figueiredo-Pereira, 2010; Arvanitakis et al., 2011). The soluble forms of Aβ may represent the primary toxic in AD. Soluble oligomers have a unique distribution in the brain of patients with AD and different types of soluble Aβ oligomers have a common structure and share a common mechanism of neurotoxicity (Kayed et al., 2003). Study has showed that soluble forms of Aβ peptide were involved in Aβ-induced cognitive impairment and caused rapid memory disruption in rats (Tucci et al., 2014). Although the exact molecular mechanism is still unknown, it is well established that Aβ-induced neuronal apoptosis triggering neuronal injury and cell loss plays a crucial role in the development of AD (Loo et al., 1993; Crews and Masliah, 2010). Studies have implicated that pro-apoptotic kinases may be a link between Aβ and tau anomalies in AD (Paquet et al., 2015). A recent report has shown that Aβ_1-42_ could cause cerebral vascular dysfunction by increasing oxidative stress and inducing mitochondrial dysfunction and apoptosis in cerebral endothelial cells in a mouse model of AD (Fang et al., 2017). Apoptosis and oxidative stress in cerebrovascular endothelial cells induced by Aβ_25-35_ promoted the development of AD (Liu et al., 2017). The blood–brain barrier (BBB) is formed by specialized brain endothelial cells and connected by extensive tight junctions (Abbott et al., 2010). The BBB plays a critical role in normal physiology of the central nervous system (CNS) and BBB dysfunction is associated with a variety of CNS diseases including brain tumors and AD (Luissint et al., 2012; Cerutti et al., 2017). Studies have shown that the BBB is responsible for Aβ exchange between the blood and the brain and it plays a vital role in controlling the concentrations of Aβ in the brain; thus, BBB dysfunction may lead to Aβ abnormal deposition in the cerebrovasculature and/or excessive accumulation in the brain (Engelhardt and Sorokin, 2009; Bachmeier et al., 2010). Evidence has shown that microvascular injury was linked to BBB leakage in AD patients (Zipser et al., 2007). Therefore, development of novel agents with anti-apoptotic activity to target human brain microvascular endothelial cells (hBMECs) may be a promising strategy for the treatment of AD.

The active ingredients from natural products such as polyphenols, flavonoids, and saponins displayed a variety of biological and neuroprotective effects and have been attracted substantial attention for the prevention and/or treatment of AD (Kim et al., 2010; Spencer, 2010; Wang et al., 2017). Previous studies have found that several compounds from natural products exhibited protective activity on vascular endothelial cells and possessed potent pharmacological effects of anti-apoptosis on hBMECs (Liu et al., 2014; Liang et al., 2017). For examples, Lutein inhibited oxidative stress induced by Aβ peptide via regulation of Nrf-2 and NF-κB in cerebrovascular endothelial cells (Liu et al., 2017). Luteolin, a natural flavonoid, has beneficial properties on the CNS and it protects the integrity of BBB by maintaining barrier function and suppressing inflammatory responses induced by fibrillary Aβ_1-40_ (Zhang J.X. et al., 2017). The mechanism of Luteolin on BBB protection may be related to the regulation of inflammatory signal pathways, such as inhibition of p38 MAPK activation, decrease of NF-κB p65 nuclear translocation, and the secretion of inflammatory cytokines (Zhang J.X. et al., 2017). *Lactobacillus rhamnosus* GG conditioned medium has protective effect on hBMECs from *Escherichia coli* K1-induced damage by inhibiting NF-κB signaling pathway (Zeng et al., 2017). Resveratrol, a phytoalexin, activates AMP-activated protein kinase (AMPK) in vascular cells. A study by Annabi et al. (2012) has shown that resveratrol prevented hBMECs dysfunction induced by neuroinflammation through inhibiting metalloproteinase (MMP)-9 and cyclooxygenase (COX)-2. Quercetin, a natural flavonoid molecule, protected hBMECs from fibrillary Aβ_1-40_-induced toxicity through alleviating intracellular reactive oxygen species (ROS) production, apoptosis and nuclear condensation as well as strengthening BBB integrity by preserving transendothelial electrical resistance (Li et al., 2015). Pinocembrin has been proved to have protective effect on microvascular function via reducing the cytotoxicity induced by fibrillar Aβ_1-40_ and inhibiting the mitogen-activated protein kinase (MAPK)/NF-κB inflammatory signaling pathways in hBMECs in AD models (Liu et al., 2014).

Asiaticoside (AS), a naturally triterpenoid saponin, isolated and extracted from Indian medicinal herb Centella asiatica (L.) Urban, displays broad bioactivities including neuroprotection, antidepressant, anti-oxidant, anti-inflammation, protection of DNA damage, and regulation of apoptotic factors in cortical neurons *in vitro* cell culture and *in vivo* animal models (Luo et al., 2015; Sun et al., 2015; Hou et al., 2016; Zhang Z. et al., 2017). The neuroprotective effects of AS have been widely reported including repairing spinal cord injury (Luo et al., 2015) and protecting neuronal damage induced by ischemia hypoxia (Sun et al., 2015). AS was able to alleviate learning and memory impairment induced by Aβ in a rat model of AD (Zhang Z. et al., 2017). Additional studies revealed that AS was capable of inhibiting several apoptotic-related signal pathways including p38-MAPK, PI3K/Akt/NF-κB, and hypoxia-induced transforming growth factor β1 (TGF-β1)/Smad2/3 (Luo et al., 2015; Wang X.B. et al., 2015; Yin et al., 2015). A recent study has shown that AS significantly inhibited tumor necrosis factor (TNF)-α induced increase in endothelial permeability through suppressing stress fiber formation (Fong et al., 2015). It is conceivable that AS possesses protective effect on hBMECs.

In the present study, we investigated the effects of AS on cytotoxicity by Cell Counting Kit-8 (CCK-8) assay; apoptosis by Hoechst 33258 staining and Annexin V-FITC (fluorescein isothiocyanate)/propidium iodide (PI) analyses; mitochondrial membrane potential by JC-1 fluorescence analysis; the protein expressions of TNF-α and IL-6 by enzyme-linked immunosorbent assay (ELISA) and TLR4, MyD88, TRAF6, p-NF-κB p65, and total NF-κB p65 by Western blotting; and nuclear translocation of NF-κB p65 by immunofluorescence analysis in hBMECs.

## Materials and Methods

### Regents

**FIGURE 1:**
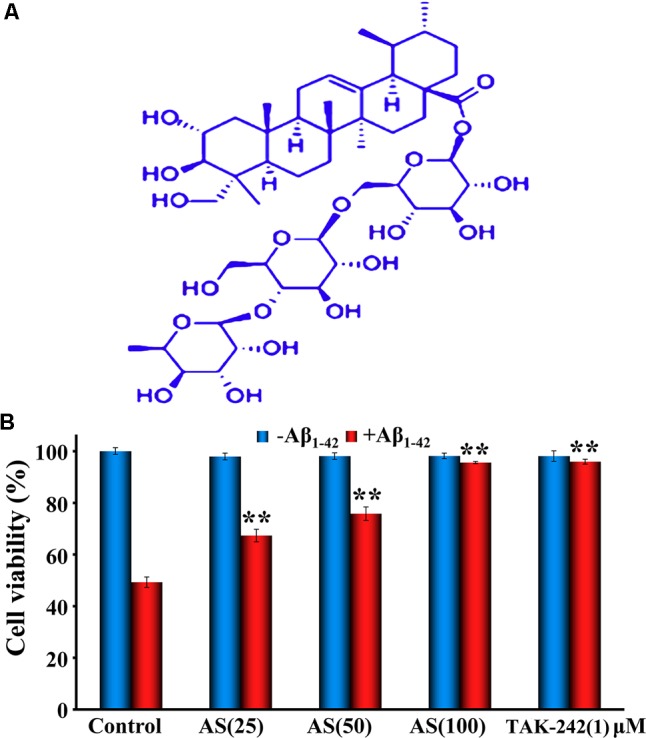
Chemical structure of asiaticoside (AS, **A**) and effects of AS and TAK-242 ± Aβ_1-42_ **(B)** on cell viability in hBMECs by CCK-8 assay. The cells were treated with AS (25, 50, and 100 μM) or TAK-242 (1 μM) for 12 h, following by Aβ_1-42_ (50 μM) or vehicle (control) for an additional 24 h. The results are representative of three independent experiments in triplicate and expressed as mean ± SD. ^∗∗^*p* < 0.01 vs. the cells treated with Aβ_1-42_ alone by two-way univariate analysis of variance (ANOVA) and Student-Newman-Keuls (SNK) test.

Synthetic Aβ_1-42_ (> 95% purity) was purchased from Sangon Biotech Company (Shanghai, China). AS (purity 98.86%, MW 959.133, **Figure 1A**) was purchased from PUSH Bio-Technology, Co., Ltd. (Chengdu, Sichuan, China). TAK-242 (resatorvid) was purchased from MedChemExpress (Monmouth Junction, NJ, United States). CCK-8 assay and Annexin V-FITC apoptosis detection kit were purchased from Dojindo Chemical Technology Co., Ltd. (Shanghai, China). All antibodies were purchased from Cell Signaling Technology Inc. (Beverly, MA, United States). Hoechst 33258 kit, JC-1 kit, Dulbecco’s modified Eagle’s medium (DMEM), dimethyl sulfoxide (DMSO), fetal bovine serum (FBS), penicillin, and streptomycin were purchased from Beyotime (Haimen, Jiangsu, China).

### Cell Culture

The hBMEC line was purchased from Procell Life Science and Technology, Co., Ltd. (Wuhan, Hubei, China). The cells were cultured in DMEM supplemented with 10% FBS, 1% penicillin and streptomycin in an atmosphere of 95% air, and 5% CO_2_ at 37°C in a humidified incubator and renewed with new medium every 3–5 days.

### Aβ_1-42_ and Drug Preparation

Synthetic Aβ_1-42_ was prepared to a final stock solution of 100 μM by dissolving in hexafluor-2-propanol (HFIP) to foster the fibrillization state. Briefly, 1 mg of Aβ_1-42_ was dissolved in 1 ml HFIP solution and distributed into centrifuge tubes after 15 min ultrasonic treatment. HFIP was removed by evaporation under vacuum at room temperature for overnight and the tubes were stored at -20°C before use. For experiments, one tube containing Aβ_1-42_ solution was taken out 24 h before experiment and resuspended in HFIP/water (ratio 3:7) for 15-min ultrasonic treatment and stirred for another 24 h; then, the solvent HFIP was evaporated under speedvac for 10 min to make 100 μM of fibrillar Aβ_1-42_ as the final solution. TAK-242 is a specific inhibitor of TLR4 receptor and used as a positive control for comparison of the effect of AS (Li et al., 2006). TAK-242 was dissolved in DMSO to a final concentration of 100 mM and stored at -20°C for future use. The concentration (1 μM) selection of TAK-242 for the present *in vitro* study was based on previous reports from the literature (Raveendran et al., 2011; Hussey et al., 2013). AS was first dissolved in methanol at a concentration of 10 mM as the stock solution and further diluted with culture medium without bovine serum to appropriate concentrations.

### Cell Viability Assay

The effects of AS and/or Aβ_1-42_ on the proliferation of hBMECs were determined by CCK-8 assay. The cells (∼80–90% confluency) were seeded in 96-well plates (1.0 ml) at a density of 5 × 10^4^ cells/well and cultured for 24 h. Then, the culture medium was replaced with serum-free DMEM and the cells were treated with different concentrations (3.125, 6.25, 12.5, 25, 50, and 100 μM) of AS or TAK-242 (1 μM) for 12 h. The vehicle solution was used as control for all experiments after it was compared to 1% methanol solution by CCK-8 assay and showed no significant difference in cell growth inhibition between the both solutions (97.00 ± 2.30% vs. 96.85 ± 3.37% in cell viability). The cells were washed with phosphate buffered solution (PBS) for 15 min at room temperature after 12 h treatment and then treated with 50 μM Aβ_1-42_ or serum-free DMEM (vehicle control) for another 24 h. After being cultured for 24 h, CCK-8 (10 μl/well) was added into the cells and incubated for 1 h. The absorbance was measured at 450 nm using SpectraMax M3 microplate readers (Molecular Devices Corporation, Sunnyvale, CA, United States). All experiments were performed three times in triplicate.

### Mitochondrial Membrane Potential (ΔψM) Detection

The changes in the mitochondrial membrane potential were determined by JC-1, a fluorescent dye with potential-dependent accumulation in mitochondrial membrane as a monomer (fluoresced green) or dimer (fluoresced red-orange) in living cells as previously described (Wu et al., 2013). Briefly, the cells were seeded at a density of 2 × 10^5^ cells/well in 6-well plates and treated with different concentrations of AS (25, 50, and 100 μM), TAK-242 (1 μM), or same volume of serum-free DMEM (vehicle control) for 12 h, then treated with Aβ_1-42_ (50 μM) or medium for another 24 h at 37°C. The cells were washed with PBS twice and incubated with JC-1 (5 μM) for 20 min at 37°C in a CO_2_ incubator, then washed with PBS three times for 5 min each time at 37°C in the dark. Fluorescent images were visualized and recorded under a fluorescence microscope at ×100 magnifications (AMG EVOS, Thermo Fisher Scientific, Inc., Waltham, MA, United States). All experiments were performed three times in triplicate.

### Apoptotic Assay of hBMECs by Hoechst 33258 Staining

Morphological assessment of apoptotic cells was processed by Hoechst 33258 staining method as described earlier (Yang et al., 2013). Briefly, hBMECs were seeded in 6-well culture plates at a density of 1 × 10^5^ cells/well and treated with various concentrations of AS (25, 50, and 100 μM), TAK-242 (1 μM), or same volume of serum-free DMEM (vehicle control) for 12 h, then treated with Aβ_1-42_ (50 μM) or medium for another 24 h at 37°C. After incubating with fixative solution for 10 min, the medium was removed and washed with PBS for 15 min. Then, the cells were treated with 500 μL staining solution of Hoechst 33258 (10 μg/ml) for 5 min, followed by washing with PBS for 15 min at a dark room for reducing the background. Finally, the nuclear morphological changes of apoptotic cells were observed under a fluorescent microscope at ×400 magnifications (AMG EVOS, Thermo Fisher Scientific, Inc., Waltham, MA, United States) and the percentage of apoptotic cells was calculated according to the ratio of apoptotic cells to total cells. All experiments were performed three times in triplicate.

### Apoptotic Assay of hBMECs with Annexin V-FITC/PI by Flow Cytometry

The cellular apoptosis of hBMECs were also determined by flow cytometry with an Annexin V-FITC apoptosis detection kit following manufacturer’s instruction. Briefly, the cells (∼80–90% confluency) were seeded in 6-well plates at a density of 2 × 10^5^ cells/well and cultured for 24 h. Then, the culture medium was replaced with serum-free DMEM and the cells were treated with AS at 25, 50, and 100 μM, TAK-242 at 1 μM, or same volume of serum-free DMEM (vehicle control) for 12 h. The cells were washed with PBS for 15 min at room temperature after 12 h treatment and treated with Aβ_1-42_ at 50 μM or same volume of medium for another 24 h. The cell suspensions were centrifuged at 1,500 × *g* for 3 min at room temperature. After centrifugation, the apoptotic cells were resuspended in Annexin V-FITC binding buffer. A total of 5 μL Annexin V-FITC and 5 μL PI were successively added to the cells and incubated for 15 min in the dark at room temperature. The quantity of stained cells was determined by flow cytometry (FACSCalibur, BD Biosciences, San Diego, CA, United States). All experiments were performed three times in triplicate.

### Analysis of TNF-α and IL-6 by ELISA

The effects of AS and/or Aβ_1-42_ on the levels of TNF-α and IL-6 in hBMECs were determined by ELISA following manufacturer’s instruction. Briefly, the cells (∼80–90% confluency) were seeded in 96-well plates at a density of 2 × 10^5^ cells/well and cultured for 24 h. Then, the culture medium was replaced with serum-free conditioned medium and the cells were treated with AS at 25, 50, and 100 μM, TAK-242 at 1 μM, or same volume of medium (vehicle control) for 12 h. The cells were washed with PBS for 15 min at room temperature after 12 h treatment and treated with Aβ_1-42_ at 50 μM or same volume of medium for another 24 h. The cell suspensions were centrifuged at 1,500 × *g* at room temperature for 5 min. Supernatants were analyzed for the levels of TNF-α and IL-6 by ELISA kit (R&D Systems, Minneapolis, MN, United States). The absorbance was determined at 450 nm using SpectraMax M3 microplate readers (Molecular Devices Corporation, Sunnyvale, CA, United States). All experiments were performed three times in triplicate.

### Western Blotting Analysis

The hBMECs were seeded at a density of 4.0 × 10^5^ cells/well on 2.0 ml 6-well plates and replaced the culture medium with serum-free DMEM after 24 h culture. Then, the cells were treated with medium (vehicle control), AS (25, 50, and 100 μM), or TAK-242 (1 μM) for 12 h. The cells were washed with PBS for 15 min at room temperature after 12 h treatment and treated with Aβ_1-42_ at 50 μM or same volume of medium for another 24 h, then, withdrawn the culture medium, and washed with cold PBS for harvest. The cell pellets were disrupted in cell lysis buffer (RIPA buffer, 0.5% NP-40, 50 mM Tris-HCl, 120 mM NaCl, 1 mM EDTA, 0.1 mM Na_3_VO_4_, 1 mM NaF, 1 mM PMSF, and 1 μg/mL leupeptin, pH 7.5), and then, the lysates were centrifuged at 9600 × *g* for 15 min at 4°C. Equal amounts of protein samples (30 μg) were electrophoresed on 12% SDS–PAGE gel for separation, followed electrophoresis, the proteins were electrotransferred from the gels to PVDF membranes to form blots. Non-specific binds were blocked with 5% skim milk in Tween-PBS buffer for 1 h, and then the membranes were incubated with anti-TLR4, anti-MyD88, anti-TRAF6, anti-NF-κB p65, and anti-NF-κB p65 (phospho S468) primary (1:1000) and secondary (1:5000) antibodies or β-actin (13E5) rabbit monoclonal antibody (1:1000) at 4°C overnight and washed three times with Tween-PBS buffer. Then, the blots were incubated for 1 h at room temperature with a 1:1000 dilution of horseradish peroxidase-labeled anti-rabbit or anti-mouse IgG (H+L) and washed three times with Tween-PBS buffer. The membranes were then developed and visualized by incubation with ECL Western detection reagents (Thermo Fisher Scientific Inc., Waltham, MA, United States). The specific protein bands were visualized and analyzed with a ChemiDoc image analyzer (Bio-Rad, Hercules, CA, United States). All experiments were performed three times in triplicate.

### Nuclear Translocation of NF-κB p65 in hBMECs by Immunofluorescence Analysis

The cells were seeded at a density of 1.0 × 10^5^ on glass coverslips and treated with medium (vehicle control), AS (25, 50, and 100 μM), or TAK-242 (1 μM) for 12 h. Then, the cells were washed with PBS for 15 min at room temperature after 12 h treatment and treated with Aβ_1-42_ (50 μM) or same volume of medium for another 24 h. The cells were fixed in 4% paraformaldehyde for 30 min at 4°C and washed with PBS for three times with 5 min for each time, then the cells were incubated with 0.1% Triton X-100 for 10 min, and blocked with 1% bovine serum albumin (BSA) for 30 min. The cells were incubated with rabbit monoclonal primary antibody (1:500) against NF-κB p65 at 4°C overnight, followed by HRP-conjugated goat anti-rabbit anti-NF-κBp65 secondary antibody (1:2000) for 2 h and washed with PBS for 15 min. The cells were stained with 4′,6-diamidino-2-phenylindole dihydrochloride (DAPI) for 10 min and washed with PBS for 15 min. The signals were detected and assessed under an inverted phase contrast fluorescence microscope (LD laser: 405 nm, 25 mW; multi-line Ar laser: 458, 488, and 515 nm, 40 mW; HeNe green laser: 543 nm, 1 mW; and HeNe red laser: 635 nm, 20 mW) at ×400 magnifications (OLYMPUS company, model: CKX41, Shinjuku-ku, Tokyo, Japan). All experiments were performed three times in triplicate.

### Statistical Analysis

All experimental data were analyzed by IBM SPSS18.0 statistical software (SPSS Inc., Chicago, IL, United States) and expressed as mean ± SD. One-way or two-way univariate analysis of variance (ANOVA) and Student-Newman-Keuls (SNK) test were performed for the analysis of variance and comparison of different groups. A difference at *p* < 0.05 was considered as statistical significance (marked as ^∗^) and higher significance level was set at *p* < 0.01 (marked as ^∗∗^).

## Results

### Protective Effect of AS on Cell Growth Inhibition Induced by Aβ_1-42_ in hBMECs

First, we evaluated the effects of AS at various concentrations (3.125, 6.25, 12.5, 25, 50, and 100 μM) and TAK-242 (1 μM) alone for 12 h exposure on cell viability of hBMECs by CCK-8 assay. The results showed that AS up to 100 μM and TAK-242 at 1 μM had no significant cell growth inhibition with the cell viability over 95% (data not shown). Next, we treated the cells with three higher concentrations (25, 50, and 100 μM) of AS and 1 μM TAK-242 for 12 h, then followed by treatment of 50 μM Aβ_1-42_ for additional 24 h. The results showed that Aβ_1-42_ (50 μM) treatment for 24 h significantly (*p* < 0.01) inhibited the cell proliferation with the cell viability of 50.89 ± 5.12% compared to 96.85 ± 3.37% in control cells treated with vehicle. However, AS treatments (25, 50, and 100 μM) significantly attenuated cell growth inhibition (*p* < 0.01) induced by Aβ_1-42_ (50 μM) in a concentration-dependent manner (**Figure 1B**). Pretreatment of TAK-242 (1 μM) also protected the cells from Aβ_1-42_-induced cell growth inhibition closed to that of 100 μM of AS (**Figure 1B**). These data suggest that AS and TAK-242 were effective to attenuate cell growth inhibition induced by Aβ_1-42_ in hBMECs.

### Effect of AS on Decrease of Mitochondrial Membrane Potential (ΔΨm) Induced by Aβ_1-42_ in hBMECs

**FIGURE 2:**
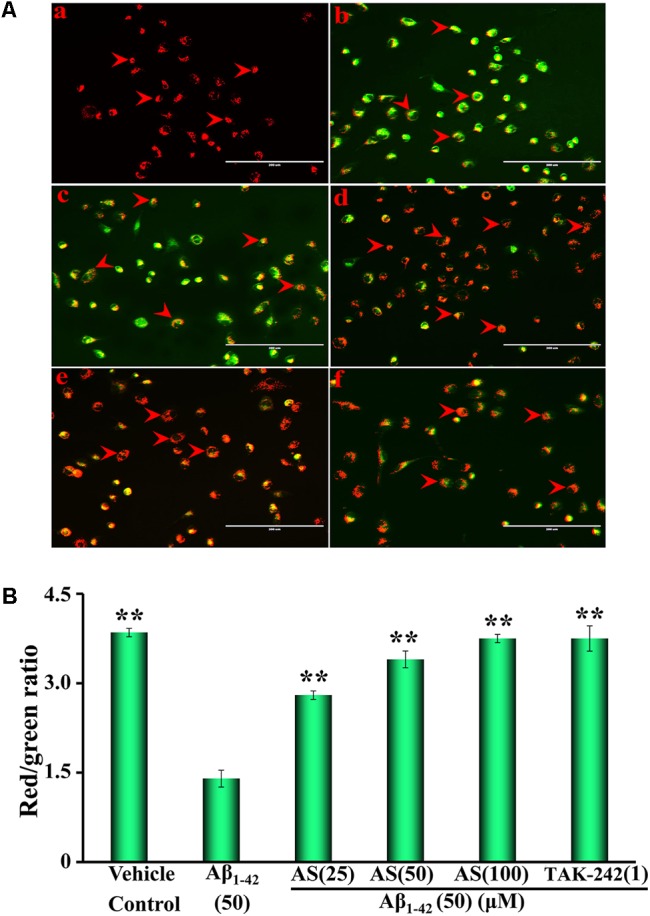
Effects of asiaticoside (AS) and TAK-242 on mitochondrial membrane potential (ΔΨm) in hBMECs. **(A)** Representative fluorescent images of mitochondrial membrane potential, the red arrows indicate apoptotic cells; **(a)** cells were treated with medium alone for 36 h; **(b)** cells were treated with medium for 12 h followed by Aβ_1-42_ 50 μM for 24 h; **(c)** cells were treated with AS 25 μM for 12 h followed by Aβ_1-42_ 50 μM for 24 h; **(d)** cells were treated with AS 50 μM for 12 h followed by Aβ_1-42_ 50 μM for 24 h; **(e)** cells were treated with AS 100 μM for 12 h followed by Aβ_1-42_ 50 μM for 24 h; **(f)** cells were treated with TAK-242 1 μM followed by Aβ_1-42_ 50 μM for 24 h. **(B)** Summarized results of red/green fluorescence ratio. The fluorescent images of mitochondrial membrane potential of hBMECs were determined by a fluorescent dye JC-1 under fluorescence microscope (×100). JC-1 accumulated in the mitochondrial membrane as fluoresced green (monomer) or fluoresced red-orange (dimer). The results are representative of three independent experiments in triplicate and expressed as mean ± SD. ^∗∗^*p* < 0.01 vs. the cells treated with Aβ_1-42_ alone by one-way univariate analysis of variance (ANOVA) and Student-Newman-Keuls (SNK) test.

Mitochondria play a critical role in cellular apoptosis and decrease of mitochondrial membrane potential is a hallmark event in the early stage of apoptosis in cells (Wlodkowic et al., 2011). Therefore, we evaluated the effects of AS (25, 50, and 100 μM) and TAK-242 (1 μM) for 12 h followed by Aβ_1-42_ (50 μM) treatment for additional 24 h on mitochondrial membrane potential (ΔΨm) in hBMECs using JC-1 fluorescence and the results are illustrated in **Figure 2**. From the representative photographs, we can see that the fluorescence intensity of red and the ratio of red/green in hBMECs were significantly (*p* < 0.01) decreased (∼2.5-fold) by Aβ_1-42_ (50 μM) treatment compared to that of the control cells treated with vehicle (**Figures 2Aa,b,B**). However, the ratio of red/green fluorescence was significantly increased (*p* < 0.01) in the cells pretreated with AS at 25, 50, or 100 μM in a concentration-dependent manner (**Figures 2Ac–e**). TAK-242 at 1 μM (**Figure 2Af**) also exhibited similar effect as 100 μM of AS on the ratio of red/green fluorescence. The data indicate that mitochondrial membrane potentials of hBMECs were significantly decreased by Aβ_1-42_ but AS and TAK-242 restored the declined ΔΨmm induced by Aβ_1-42_ (**Figure 2B**), therefore to inhibit Aβ_1-42_-induced apoptosis via maintaining high mitochondrial membrane potential.

### Effect of AS on Aβ_1-42_-Induced Apoptosis in hBMECs by Hoechst 33258 Staining and Annexin V/PI Double Staining Assays

The crucial factor for pathogenesis of AD is neuronal cell damage and loss due to Aβ-induced apoptosis (Loo et al., 1993; Crews and Masliah, 2010). Therefore, we studied the protective effect of AS on apoptosis induced by Aβ_1-42_ in hBMECs.

**FIGURE 3:**
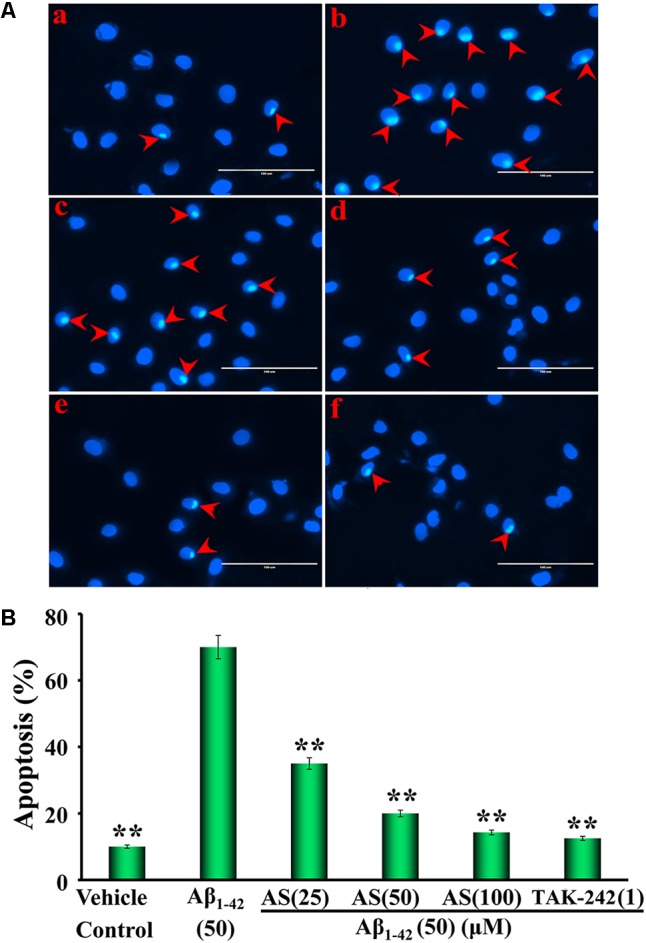
Effects of asiaticoside (AS) and TAK-242 on apoptosis induced by Aβ_1-42_ in hBMECs with Hoechst 33258 staining under fluorescence microscope (×400). **(A)** Representative photographs of apoptotic cells indicated by red arrows; **(a)** cells were treated with medium alone for 36 h; **(b)** cells were treated with medium for 12 h followed by Aβ_1-42_ 50 μM for 24 h; **(c)** cells were treated with AS 25 μM for 12 h followed by Aβ_1-42_ 50 μM for 24 h; **(d)** cells were treated with AS 50 μM for 12 h followed by Aβ_1-42_ 50 μM for 24 h; **(e)** cells were treated with AS 100 μM for 12 h followed by Aβ_1-42_ 50 μM for 24 h; **(f)** cells were treated with TAK-242 1 μM followed by Aβ_1-42_ 50 μM for 24 h. **(B)** Summarized results of percentage (%) of apoptosis. The results are representative of three independent experiments in triplicate and expressed as mean ± SD. ^∗∗^*p* < 0.01 vs. the cells treated with Aβ_1-42_ alone by one-way univariate analysis of variance (ANOVA) and Student-Newman-Keuls (SNK) test.

Hoechst 33258 staining is a classical and rapid method for the detection of apoptosis by observation of chromatin condensation under a fluorescence microscopy (Yang et al., 2013). The representative morphological photographs of hBMECs and the cell apoptotic ratio by Hoechst 33258 staining method are illustrated in **Figure 3**. The photograph in **Figure 3Aa** shows the characteristic preserved normal features of hBMECs in the control cells without Aβ_1-42_ treatment, which the cell nuclei appear blue fluorescence. However, higher levels of apoptotic cells with condensation of nuclear chromatin and fragmentation stained with white color were detected in the cells treated with Aβ_1-42_ (50 μM) for 24 h (**Figure 3Ab**). Interestingly, the numbers of apoptotic cells were markedly decreased with pretreatment of AS (25, 50, and 100 μM) or TAK-242 (1 μM) for 12 h (**Figures 3Ac–f**). The summarized results of apoptotic ratio of cells are shown in **Figure 3B** and indicate that Aβ_1-42_ (50 μM) treatment significantly increased apoptosis (over 70% apoptotic cells) compared to that of control cells treated with vehicle (less than 10% apoptotic cells, *p* < 0.01), while pretreatment of AS significantly inhibited Aβ_1-42_-induced apoptosis in a concentration-dependent manner (*p* < 0.01) in hBMECs. In additional TAK-242 (1 μM) exhibited inhibitory effect on Aβ_1-42_-induced apoptosis as more potent than that of 50 μM but less than that of 100 μM of AS.

**FIGURE 4:**
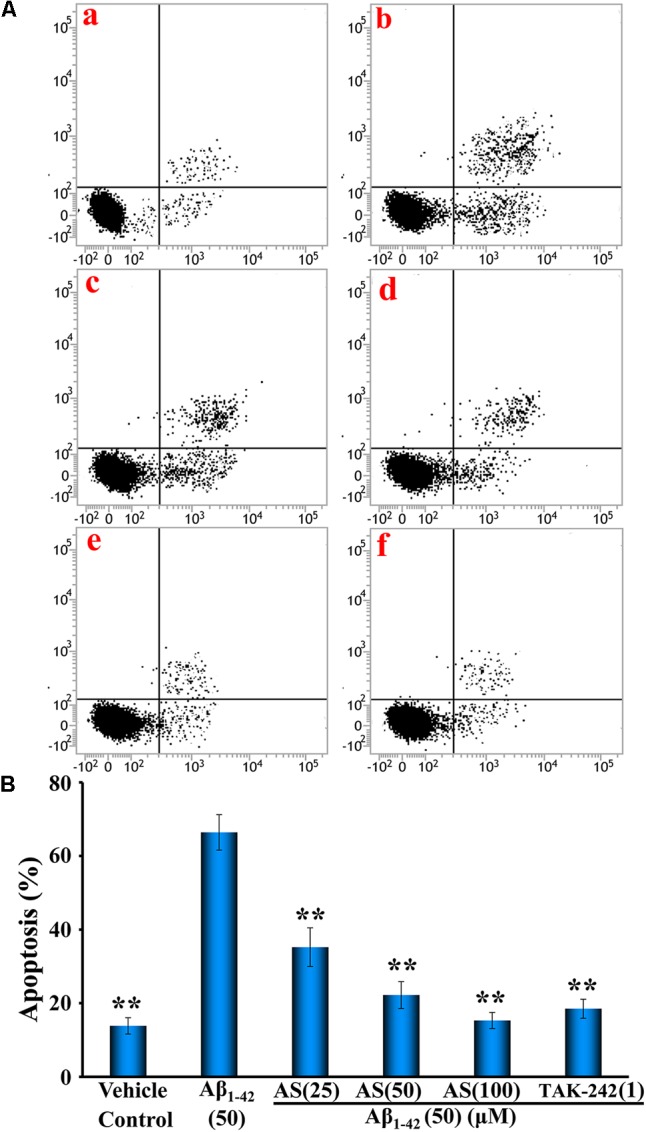
Effects of asiaticoside (AS) and TAK-242 on apoptosis induced by Aβ_1-42_ in hBMECs with Annexin V-FITC/PI staining by flow cytometry analysis. **(A)** Representative pictures of apoptotic cells by Annexin V-FITC/PI staining; **(a)** cells were treated with medium alone for 36 h; **(b)** cells were treated with medium for 12 h followed by Aβ_1-42_ 50 μM for 24 h; **(c)** cells were treated with AS 25 μM for 12 h followed by Aβ_1-42_ 50 μM for 24 h; **(d)** cells were treated with AS 50 μM for 12 h followed by Aβ_1-42_ 50 μM for 24 h; **(e)** cells were treated with AS 100 μM for 12 h followed by Aβ_1-42_ 50 μM for 24 h; **(f)** cells were treated with TAK-242 1 μM followed by Aβ_1-42_ 50 μM for 24 h. **(B)** Summarized results of percentage (%) of apoptosis. The results are representative of three independent experiments in triplicate and expressed as mean ± SD. ^∗∗^*p* < 0.01 vs. the cells treated with Aβ_1-42_ alone by one-way univariate analysis of variance (ANOVA) and Student-Newman-Keuls (SNK) test.

Annexin V is labeled with fluorescein (FITC) as fluorescent probe and one of the sensitive methods to detect the early apoptosis of cells (Wlodkowic et al., 2011). Therefore, we further investigated the quantity of apoptotic cells induced by Aβ_1-42_ and the protective effects of AS (25, 50, and 100 μM) and compared to that of TAK-242 (1 μM) with Annexin V/PI double staining by flow cytometric analysis and the results are illustrated in **Figure 4**. The data showed that 24 h treatment of Aβ_1-42_ (50 μM) significantly induced apoptosis in hBMECs to 66.40 ± 4.82% compared to 13.82 ± 2.22 % (*p* < 0.01) in control cells treated with vehicle (**Figures 4Aa,b**). Interestingly, pretreatment of AS at 25, 50, and 100 μM significantly decreased apoptotic cells (*p* < 0.01) induced by Aβ_1-42_ (50 μM) compared to Aβ_1-42_ alone to 35.20 ± 5.26%, 22.20 ± 3.66%, and 15.28% ± 2.18% in hBMECs, respectively (**Figures 4Ac–e,B**). Pretreatment of TAK-242 (50 μM) also significantly inhibited Aβ_1-42_-induced apoptosis with 18.48 ± 2.58% in hBMECs (**Figures 4Af,B**). The results clearly demonstrated that AS effectively inhibited Aβ_1-42_-induced apoptosis in a concentration-dependent manner in hBMECs by both methods of Hoechst 33258 Staining and Annexin V/PI Double Staining with flow cytometric analysis and indicate that AS and TAK-242 have protective effect on hBMECs cells from β_1-42_-induced apoptosis.

### AS Downregulates Inflammatory Cytokines of TNF-α and IL-6 Induced by Aβ_1-42_ in hBMECs

The TNF-α is one of the most important inflammatory mediators and appears in the earliest process of apoptosis (Wang W.Y. et al., 2015). IL-6 is a key pro-inflammatory cytokine and plays an important role in inflammation and immune response of the body as well as in the regulation of metabolic, regenerative, and neural processes (Scheller et al., 2011). Therefore, we evaluated the effect of AS on the levels of TNF-α and IL-6 induced by Aβ_1-42_ and compared to that of TAK-242 in hBMECs by ELISA analysis.

**FIGURE 5:**
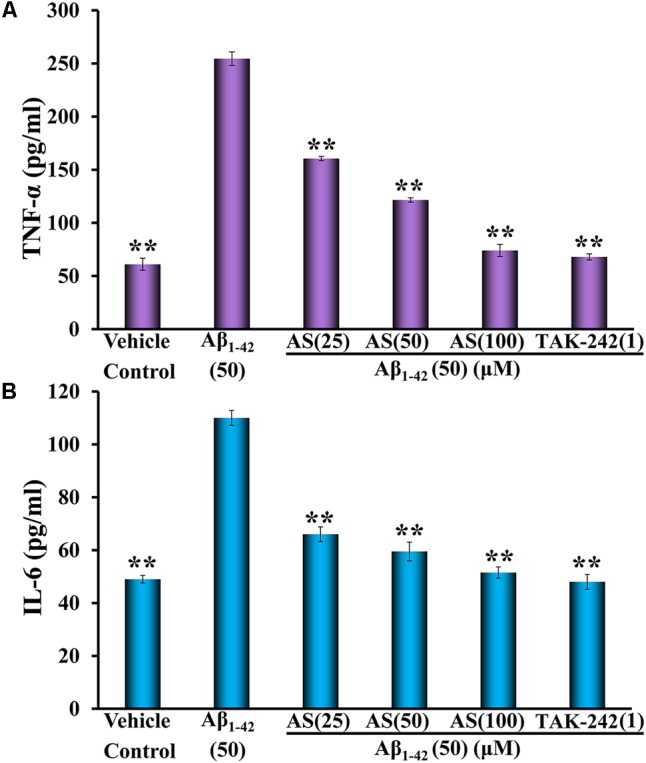
Effects of asiaticoside (AS) and TAK-242 on inflammatory cytokines of TNF-α **(A)** and IL-6 **(B)** induced by Aβ_1-42_ in hBMECs detected by ELISA. The cells were treated with medium (vehicle control), AS (25, 50, and 100 μM), or TAK-242 (1 μM) for 12 h followed by Aβ_1-42_ (50 μM) or medium for 24 h. The results are representative of three independent experiments in triplicate and expressed as mean ± SD. ^∗∗^*p* < 0.01 vs. the cells treated with Aβ_1-42_ alone by one-way univariate analysis of variance (ANOVA) and Student-Newman-Keuls (SNK) test.

The expressions of TNF-α and IL-6 were significantly increased (*p* < 0.01) by Aβ_1-42_ (50 μM) treatment compared to vehicle treatment in hBMECs (**Figure 5**). However, pretreatment of AS at 25, 50, and 100 μM significantly decreased the elevated expressions of TNF-α and IL-6 induced by Aβ_1-42_ in hBMECs (**Figure 5**). TAK-242 at 1 μM had similar effect as 100 μM of AS on decreasing the elevated expressions of TNF-α and IL-6 induced by Aβ_1-42_ in hBMECs (**Figure 5**). These results suggest that AS and TAK-242 effectively inhibited Aβ_1-42_-induced high expressions of TNF-α and IL-6 and the effects of AS and TAK-242 on decrease of apoptosis induced by Aβ_1-42_ may be via inhibiting the expressions of TNF-α and IL-6 in hBMECs.

### Effect of AS on Inhibition of TLR4/NF-κB Signaling Pathway in hBMECs

**FIGURE 6:**
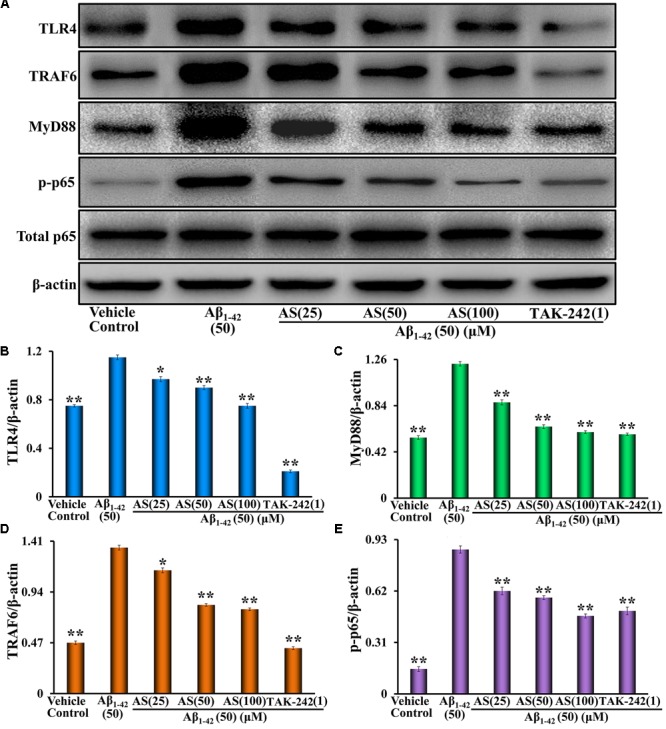
Effects of asiaticoside (AS) and TAK-242 on the protein expressions of TLR4, MyD88, TRAF6, p-NF-κB p65, and total NF-κB p65 induced by Aβ_1-42_ in hBMECs by Western blotting analysis. **(A)** The bands of TLR4, MyD88, TRAF6, p-NF-κB p65, total NF-κB p65, and β-actin. β-actin was used as a loading control. **(B)** The protein expression of TLR4; **(C)** The protein expression of MyD88; **(D)** The protein expression of TRAF6; and **(E)** the protein expression of p-NF-κB p65. The cells were treated with medium (vehicle control), AS (25, 50, and 100 μM), or TAK-242 (1 μM) for 12 h followed by Aβ_1-42_ (50 μM) or medium for 24 h. The results are representative of three independent experiments in triplicate and expressed as mean ± SD. ^∗∗^*p* < 0.01 vs. the cells treated with Aβ_1-42_ alone by one-way univariate analysis of variance (ANOVA) and Student-Newman-Keuls (SNK) test.

The TLR4/NF-κB signaling pathway plays an important role in the regulation of cell apoptosis (Liu et al., 2013). NF-κB signaling activation involves phosphorylation and nuclear translocation of the p65 protein (Christian et al., 2016). We speculated that the anti-apoptotic effect of AS may be related to inhibition of the expressions of proteins in TLR4/NF-κB signaling pathway and translocation of NF-κB p65 in hBMECs. Therefore, we evaluated the effects of AS on the expressions of TLR4, MyD88, TRAF6, p-NF-κB p65, and total NF-κB p65 proteins and NF-κB p65 translocation. We treated hBMECs with medium (vehicle control), AS (25, 50, and 100 μM) or TAK-242 (1 μM) for 12 h followed by Aβ_1-42_ (50 μM) treatment for additional 24 h and analyses by Western blotting and Immunofluorescence assay. The results showed that Aβ_1-42_ treatment significantly upregulated the protein expressions of TLR4, MyD88, TRAF6, and p-NF-κB p65 (*p* < 0.01) but no effect on total NF-κB p65 compared to vehicle treatment in hBMECs (**Figure 6**). However, pretreatment of AS significantly (*p* < 0.01 vs. Aβ_1-42_ treated group) downregulated the highly elevated levels of TLR4, MyD88, TRAF6, and p-NF-κB p65 induced by Aβ_1-42_ in hBMECs (**Figure 6**). Pretreatment of TAK-242, a specific TLR4 inhibitor, also significantly decreased (*p* < 0.01) the protein expressions of TLR4, MyD88, TRAF6, and p-NF-κB p65 being upregulated by Aβ_1-42_ in hBMECs (**Figure 6**).

**FIGURE 7:**
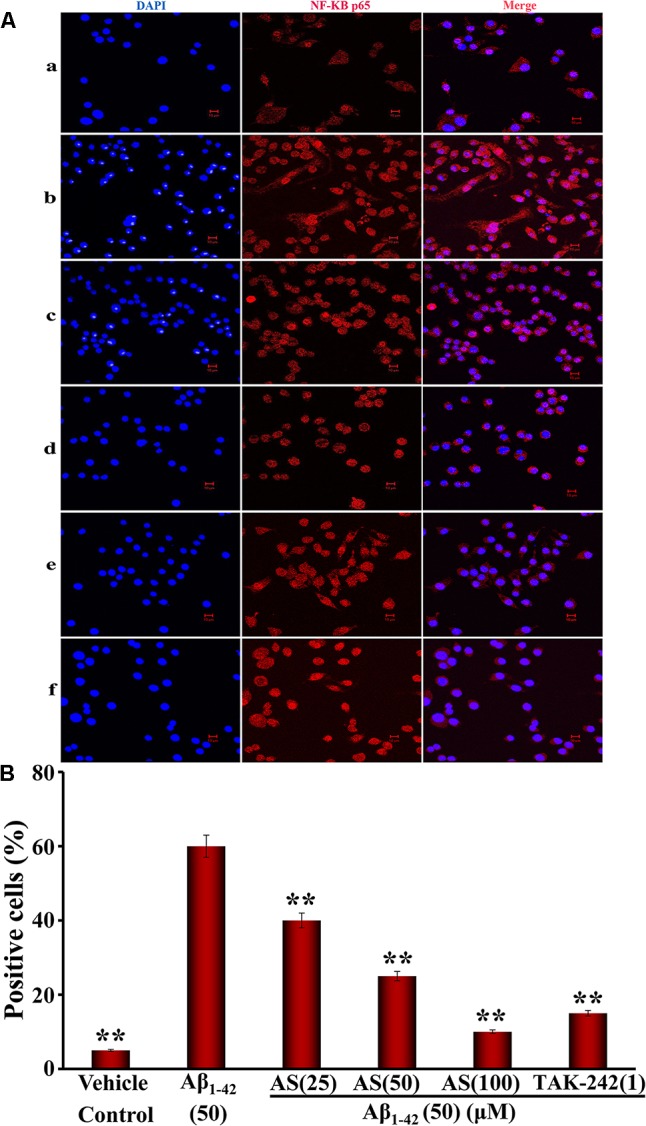
Effects of asiaticoside (AS) and TAK-242 on nuclear translocation of NF-κB p65 induced by Aβ_1-42_ in hBMECs by immunofluorescence analysis (×400). **(A)** Representative pictures of nuclear translocation of NF-κB p65 by immunofluorescence analysis; **(a)** cells were treated with medium alone for 36 h; **(b)** cells were treated with medium for 12 h followed by Aβ_1-42_ 50 μM for 24 h; **(c)** cells were treated with AS 25 μM for 12 h followed by Aβ_1-42_ 50 μM for 24 h; **(d)** cells were treated with AS 50 μM for 12 h followed by Aβ_1-42_ 50 μM for 24 h; **(e)** cells were treated with AS 100 μM for 12 h followed by Aβ_1-42_ 50 μM for 24 h; **(f)** cells were treated with TAK-242 1 μM followed by Aβ_1-42_ 50 μM for 24 h. DAPI: blue channel; NF-κB p65: red channel; and Merge: combination of red and blue channels. **(B)** Summarized results of percentage (%) of positive cells with nuclear translocation of NF-κB p65. The results are representative of three independent experiments in triplicate and expressed as mean ± SD. ^∗∗^*p* < 0.01 vs. the cells treated with Aβ_1-42_ alone by one-way univariate analysis of variance (ANOVA) and Student-Newman-Keuls (SNK) test.

Next, we determined the localization of NF-κB p65 in hBMECs. Aβ_1-42_ (50 μM) treatment resulted in a significant translocation of p65 from a cytoplasmic to a nuclear localization compared to that of vehicle treatment (p65 mainly located in the cytoplasm) in hBMECs (**Figures 7Aa–b,B**). However, pretreatment of AS (25, 50, and 100 μM) significantly inhibited the nuclear translocation of p65 in a concentration-dependent manner compared to Aβ_1-42_ treated group (*p* < 0.01) in hBMECs (**Figures 7Ac–e,B**). Similarly, pretreatment of TAK-242 (1 μM) also significantly (*p* < 0.01) inhibited the nuclear translocation of p65 (**Figures 7Af,B**). The results indicate that AS and TAK-242 effectively inhibited Aβ_1-42_-induced high expressions of TLR4, MyD88, TRAF6, and p-NF-κB p65 proteins and NF-κB p65 nuclear translocation. Therefore, the effects of AS and TAK-242 on inhibition of apoptosis induced by Aβ_1-42_ may be through suppressing TLR4/NF-κB signaling pathway in hBMECs.

## Discussion

Centella asiatica (L.) Urban is a dicotyledonous plant of Umbelliferae centella dry grass or whole plant with roots (Lin et al., 2017). Numerous studies have shown that centella asiatica possesses prominent pharmacological effects including memory improvement, anti-cancer, and cardiovascular protection (Mannangatti and Naidu, 2016; Yang et al., 2016; Wu et al., 2017). AS is a main constituent isolated and extracted from centella and it could improve memory ability of rats with cognitive impairment induced by injection of Aβ into the brain (Mook-Jung et al., 1999). AS also displayed protective effect on Aβ_1-42_-induced apoptosis in PC12 cells and reduced acute lung injury induced by lipopolysaccharide in mice (Qiu et al., 2015; Zhang et al., 2015; Zhang Z. et al., 2017).

It is worth noting that we previously reported that AS could attenuate neurotoxic effects of Aβ in hBMECs *in vitro* (Zhang et al., 2015) and improve learning and memory function in a rat model of AD *in vivo* (Zhang Z. et al., 2017), however, the underlying molecular mechanism of the findings remains unclear and needs to be further elucidated. We hypothesize that AS alleviates Aβ_1-42_-induced apoptosis through inhibiting the TLR4/NF-κB signaling pathway in hBMECs, and AS may be potentially developed as a novel agent for the prevention and treatment of patients with AD. Therefore, we attempted to elucidate the molecular mechanism of the protective effect of AS on hBMECs. Here, we showed that AS treatment could inhibit Aβ_1-42_-induced cytotoxicity and apoptosis in hBMECs (**Figures 1, 3, 4**). It thus seems plausible that the protective effect of AS on cell growth inhibition induced by Aβ_1-42_ may be through suppression of apoptosis. The crucial factor for the pathogenesis of AD is neuronal cell damage and loss due to Aβ-induced apoptosis (Loo et al., 1993; Crews and Masliah, 2010). Therefore, AS would be effective in the prevention and treatment of AD. However, how and to what degree of the effects of AS on the hBMECs and the relationship for its brain protection and anti-apoptosis in brain cells as well as other possible mechanisms beside apoptosis are seriously challenging and remain to be further investigated with appropriate experiments.

Our results showed that AS itself up to 100 μM had little or no toxic to hBMECs. However, treatment of Aβ_1-42_ at 50 μM for 24 h produced significant cytotoxicity (∼50% cell viability), while AS and TAK-242 significantly protected hBMECs from Aβ_1-42_-induced cytotoxicity (**Figure 1B**). The results are consistent with previous reports of pharmacological studies of AS (Qiu et al., 2015; Zhang et al., 2015; Zhang Z. et al., 2017; Mannangatti and Naidu, 2016; Yang et al., 2016; Wu et al., 2017). Additionally, our results showed that pretreatment of AS could obviously attenuate apoptosis induced by Aβ_1-42_ in hBMECs (**Figures 3, 4**).

Mitochondria play a key role in the progression of apoptosis and mitochondrial membrane potential is decreased at the early stage of cellular apoptosis (Wlodkowic et al., 2011). It is easy to detect the decrease of cell membrane potential with JC-1 when the red fluorescence transforms to green fluorescence and JC-1 change is also regarded as a marker for early apoptosis (Wlodkowic et al., 2011). Our results demonstrated that mitochondrial membrane potentials of hBMECs were significantly decreased by Aβ_1-42_ but pretreatment of AS and TAK-242 could restore the declined mitochondrial membrane potentials induced by Aβ_1-42_ (**Figure 2**). Therefore, the effects of AS in inhibiting Aβ_1-42_-induced apoptosis may be, at least in part, via maintaining high mitochondrial membrane potential of hBMECs. Aβ-induced apoptosis is the key factor for pathogenesis of AD by induced neuronal cell damage and loss (Loo et al., 1993; Crews and Masliah, 2010). Therefore, we evaluated the effect of AS on apoptosis in hBMECs by Hoechst 33258 staining and Annexin V-FITC/PI analysis (**Figures 3, 4**). Hoechst 33258 staining is a classical and rapid detection method for cell apoptosis via observing chromatin condensation under a fluorescence microscopy (Yang et al., 2013). Annexin V-FITC/PI is one of the sensitive methods for early apoptosis detection of cells via fluorescent probe (Wlodkowic et al., 2011). Phosphatidylserine is mainly distributed in the membrane lipid bilayer inside in normal cells, which can transform from inside to outside on membrane in the early stage of apoptosis. Annexin V is a Ca^2+^-dependent phospholipid-binding protein with high affinity for phosphatidylserine, which adheres to the membrane of early apoptotic cells by binding to phosphatidylserine on the outside of cells (Demchenko, 2013). Our data showed that Aβ_1-42_ treatment (50 μM) for 24 h obviously increased apoptosis (∼70% apoptotic cells) compared to that of control cells treated with vehicle (∼10–15% apoptotic cells, *p <* 0.01), while pretreatment of AS (25, 50, and 100 μM) significantly inhibited Aβ_1-42_-induced apoptosis in a concentration-dependent manner compared to Aβ_1-42_ treatment alone (*p* < 0.01) in hBMECs with both methods (**Figures 3, 4**).

The TNF-α is an earliest and most important inflammatory mediator and appears in the process of apoptosis (Wang W.Y. et al., 2015). TNF-α activates neutrophils and lymphocytes, increases the permeability of vascular endothelial cells, regulates metabolic activity of tissues, and promotes synthesis and release of other cytokines (Duque and Descoteaux, 2014). TNF-α also could induce phosphorylation of NF-κB p65 protein at T254 (Christian et al., 2016). IL-6 induces B cells differentiation to produce antibodies and also induces the activation and proliferation of T cells in the immune response of the body (Duque and Descoteaux, 2014). IL-6 is a key pro-inflammatory cytokine and plays an important role in inflammation and immune response (Scheller et al., 2011). IL-6 also has numerous activities outside of the immune system such as regulation of metabolic, regenerative, and neural processes (Scheller et al., 2011). Therefore, we evaluated the effect of AS on the levels of TNF-α and IL-6 in hBMECs induced by Aβ_1-42_. The expressions of TNF-α and IL-6 in hBMECs were significantly increased (*p* < 0.01) by Aβ_1-42_ (50 μM) treatment compared to vehicle treatment (**Figure 5**). However, AS (25, 50, and 100 μM) significantly decreased the elevated expressions of TNF-α and IL-6 induced by Aβ_1-42_ in hBMECs.

The TLR4/NF-κB signaling pathway plays an important role in the regulation of cell proliferation and apoptosis (Liu et al., 2013). Studies have proved that activated NF-κB promotes cell apoptosis and some types of NF-κB subunits play a crucial role in cell apoptosis (Chen et al., 2001; Luo et al., 2005; Christian et al., 2016; Park and Hong, 2016). The activated NF-κB translocates from cytoplasm into nucleus of cell to regulate gene expressions of cytokines and to promote protein synthesis, finally, leads to cell apoptosis (Wan and Lenardo, 2010). Furthermore, phosphorylation plays a critical role in the activation of NF-κB downstream and the phosphorylation of its subunits has a profound effect on its function (Christian et al., 2016). So far, p65 has received the most attention in NF-κB phosphorylation among the NF-κB subunits. The activation of NF-κB signaling pathway involves phosphorylation and nuclear translocation and retention of p65 to promote gene expression and regulate transcriptional activity (Christian et al., 2016). Therefore, target of TLR4/NF-κB signaling pathway may provide a new strategy for the treatment of human common diseases such as inflammatory disease, neurodegenerative disease, and cancer. The present studies showed that Aβ_1-42_ (50 μM) treatment for 24 h significantly increased the protein expressions of TLR4, MyD88, TRAF6, and p-NF-κB p65 compared to vehicle treatment (*p* < 0.01) in hBMECs (**Figure 6**). However, pretreatment of AS (25, 50, and 100 μM) for 12 h significantly downregulated (*p* < 0.01 vs. Aβ_1-42_ treated group) the highly elevated levels of TLR4, MyD88, TRAF6, and p-NF-κB p65 proteins induced by Aβ_1-42_ in hBMECs (**Figure 6**). Furthermore, we determined the localization of NF-κB p65 in hBMECs and the results showed that NF-κB p65 was mainly located in the cytoplasm in the cells treated with vehicle control (**Figure 7Aa**), but Aβ_1-42_ (50 μM) treatment for 24 h resulted in a significant translocation of p65 from cytoplasm to nucleus (**Figure 7Ab**). However, pretreatment of AS (25, 50, and 100 μM) for 12 h could reverse the effect of p65 nuclear translocation induced by Aβ_1-42_ treatment (**Figures 7Ac–e,B**). In additional, similar effects of downregulation of TLR4, MyD88, TRAF6, and p-NF-κB p65 proteins as well as inhibition of p65 nuclear translocation were also observed with the specific TLR4 inhibitor of TAK-242 (**Figures 6, 7**). Previous reports have shown that MyD88 and TRAF6 are the key adaptors of TLR4 signaling pathway and play crucial role in the pathway (Laird et al., 2009; Zhou et al., 2010). Therefore, these results indicate that inhibition of Aβ_1-42_-induced cell apoptosis by AS may be through suppressing the TLR4/NF-κB signaling pathway.

Other protective effects of AS including its anti-oxidative activity may also contribute to its protective effect on Aβ_1-42_-induced damage in hBMECs (Xing et al., 2017), although we did not evaluate its effect on reduction of oxidative stress induced by Aβ_1-42_ in hBMECs in the present studies. The present studies were mainly focused on extending and validating our previous findings of AS in inhibition of Aβ_1-42_-induced apoptosis and further determined the associated mechanism in hBMECs (Zhang et al., 2015).

**FIGURE 8:**
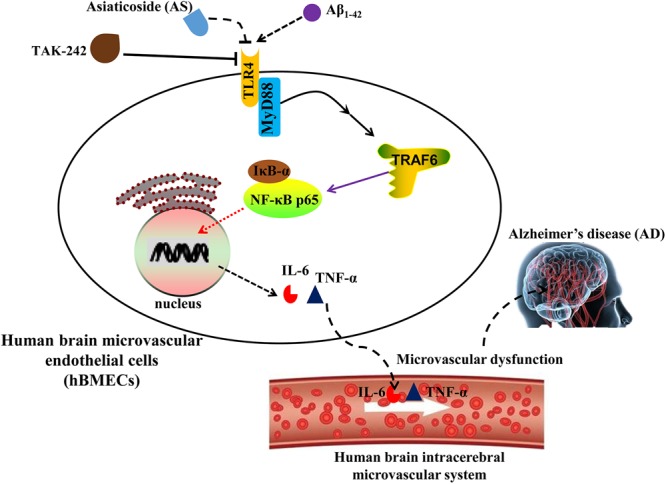
Proposed scheme of the possible mechanisms associated with the effects of asiaticoside (AS) on attenuation of cell growth inhibition and apoptosis induced by Aβ_1-42_ in human brain microvascular endothelial cells (hBMECs).

The proposed possible mechanisms for the protective effects of AS on Aβ_1-42_-induced cytotoxicity and apoptosis in hBMECs are summarized in **Figure 8**. However, more investigations of other cellular signaling pathways are needed to be done for the complexity of the molecular mechanisms associated with the effects of AS. Therefore, our further studies into the molecular mechanisms associated with the effect of AS on cell growth inhibition and anti-apoptosis should include the upstream regulation of PI3K/Akt and apoptotic pathways and its effect on anti-oxidative activity in hBMECs. Furthermore, the *in vitro* findings of AS are needed to be validated by *in vivo* studied of animal models and further by clinical trials.

## Conclusion

The data from the present studies showed that AS could attenuate cytotoxicity and apoptosis, and restore declined mitochondrial membrane potential induced by Aβ_1-42_ in hBMECs. AS also significantly downregulated the highly elevated expressions of TNF-α, IL-6, TLR4, MyD88, TRAF6, and p-NF-κB p65, as well as inhibited NF-κB p65 translocation from cytoplasm to nucleus induced by Aβ_1-42_ in a concentration-dependent manner in hBMECs. The possible underlying molecular mechanism of AS may be through inhibiting the TLR4/NF-κB signaling pathway. Therefore, AS has the potential to be developed as a novel agent for the prevention and/or treatment of AD clinically. However, other possible molecular mechanisms associated with the effects of AS such as anti-oxidative activity and upstream regulation of PI3K/Akt and apoptotic pathways as well as *in vivo* study of animal models are needed to be further investigated and validated by clinical trials.

## Author Contributions

ZZ and SC designed the experiments and analyzed the data. DS, XJ, YL, and YS performed the experiments. DS, ZZ, and SC wrote the manuscript. All authors discussed the results and contributed to the manuscript.

## Conflict of Interest Statement

The authors declare that the research was conducted in the absence of any commercial or financial relationships that could be construed as a potential conflict of interest.
